# Regeneration of functional alveoli by adult human SOX9^+^ airway basal cell transplantation

**DOI:** 10.1007/s13238-018-0506-y

**Published:** 2018-01-17

**Authors:** Qiwang Ma, Yu Ma, Xiaotian Dai, Tao Ren, Yingjie Fu, Wenbin Liu, Yufei Han, Yingchuan Wu, Yu Cheng, Ting Zhang, Wei Zuo

**Affiliations:** 1 Shanghai Pulmonary Hospital, School of Medicine, Tongji University, Shanghai, 200433 China; 2 Southwest Hospital, Third Military Medical University of PLA, Chongqing, 400038 China; 3 Shanghai Jiao Tong University Affiliated Sixth People’s Hospital, Shanghai, 200233 China; 4 The Institute for Biomedical Engineering and Nano Science, School of Medicine, Tongji University, Shanghai, 200029 China; 5 Kiangnan Stem Cell Institute, Zhejiang, 311300 China; 6 Guangzhou Institute of Respiratory Disease, The First Affiliated Hospital of Guangzhou Medical University, Guangzhou, 510120 China

**Keywords:** lung, regeneration, transplantation, stem cell, bronchiectasis, alveoli

## Abstract

Irreversible destruction of bronchi and alveoli can lead to multiple incurable lung diseases. Identifying lung stem/progenitor cells with regenerative capacity and utilizing them to reconstruct functional tissue is one of the biggest hopes to reverse the damage and cure such diseases. Here we showed that a rare population of SOX9^+^ basal cells (BCs) located at airway epithelium rugae can regenerate adult human lung. Human SOX9^+^ BCs can be readily isolated by bronchoscopic brushing and indefinitely expanded in feeder-free condition. Expanded human SOX9^+^ BCs can give rise to alveolar and bronchiolar epithelium after being transplanted into injured mouse lung, with air-blood exchange system reconstructed and recipient’s lung function improved. Manipulation of lung microenvironment with Pirfenidone to suppress TGF-β signaling could further boost the transplantation efficiency. Moreover, we conducted the first autologous SOX9^+^ BCs transplantation clinical trial in two bronchiectasis patients. Lung tissue repair and pulmonary function enhancement was observed in patients 3–12 months after cell transplantation. Altogether our current work indicated that functional adult human lung structure can be reconstituted by orthotopic transplantation of tissue-specific stem/progenitor cells, which could be translated into a mature regenerative therapeutic strategy in near future.

**Electronic supplementary material:**

The online version of this article (10.1007/s13238-018-0506-y) contains supplementary material, which is available to authorized users.

## Introduction

Regeneration of human skin (Gallico et al., 1984), corneal epithelium (Rama et al., 2001; Rama et al., 2010) and hematopoietic system (Copelan, 2006) by autologous transplantation of tissue-specific stem/progenitor cell has been achieved decades ago and now has become a routine therapeutic approach. However, regeneration of large inner organs, such as lung, remains one of the biggest challenges to modern medicine. Lung-related diseases are the third-leading cause of human death globally. Most of the lethal lung diseases such as chronic obstructive pulmonary disease (COPD) (Mannino, 2002), idiopathic pulmonary fibrosis (Selman et al., 2004) and bronchiectasis (Moulton and Barker, 2012) are characterized by irreversible, progressive damage of lung tissues (alveoli and/or bronchi). Besides the mitigating treatments available, lung transplant surgery is the only solution for the exacerbated patients but its application is largely limited due to the extreme lack of donor lung as well as severe side effects resulted from immune-rejection. As a potential substitute, the transplantable artificial lung technique is promising but still in its infancy (Ott et al., 2010). Therefore, millions of patients are in urgent need of a new strategy to cure such diseases and stem/progenitor cell-based regenerative therapy is likely to be the biggest hope for them. Among all cells with clinical potential, mesenchymal stem cells (MSCs) or other stroma-derived cells are easy to obtain and handle. However, it is widely recognized that transplanted MSCs function mainly through paracrine or immunomodulatory mechanism (Meirelles Lda et al., 2009), with no evidence showing that they can reconstitute lung structure for regeneration purpose. Induced pluripotent stem cells (iPSCs) could be another source of “self” stem cells for autologous transplantation and indeed, iPSCs have been successfully coaxed to alveolar and airway lineage *in vitro* (Huang et al., 2014). However the capability of iPSC-derived cells to generate real lung structure and their tumorigenic risk remains to be evaluated *in vivo* (Kotton and Morrisey, 2014). To this end, tissue-resident progenitor cells from an adult’s own lung—if can be identified, isolated and expanded—can be a new option for transplantation therapy.

In adult rodent, different populations of lung stem/progenitor cells have been identified in last decade with capability to reconstruct lung epithelium. Most of the mouse lung stem/progenitor cells are facultative and can be induced to proliferate in response to injury as well as differentiate into one or more lung cell types (Kotton and Morrisey, 2014; Kim et al., 2005; Barkauskas et al., 2013; Hogan et al., 2014; Desai et al., 2014). More recently, we and others found a rare population of p63^+^/Krt5^+^ distal airway stem cells (DASCs), which play essential role in murine lung repair after influenza-induced acute injury (Zuo et al., 2015; Vaughan et al., 2015). However in adult human, whether there are lung cells with regenerative capacity *in vivo* need to be explored. Given the huge differences between human vs. mouse of their respiratory systems in terms of developmental process, lung lobulation, branching pattern and cell composition, the identity of human lung progenitor cells need to be rigorously evaluated.

In the current work, we discovered the putative adult human lung progenitor cells located at the bottom of “rugaes” in airway epithelium, with a SOX9 marker to distinguish them from other SOX9^−^/P63^+^/KRT5^+^ airway basal cells (BCs). From a trace amount of bronchoscopic brush-off lung tissues, we isolated SOX9^+^ BCs and expanded them *in vitro* indefinitely. SOX9^+^ BCs transplanted into injured immune-deficient mouse lung can regenerate functional lung epithelium with both human bronchiolar and alveolar epithelium reconstituted. Most importantly, for the first time we explored the clinical feasibility of autologous SOX9^+^ BC transplantation to treat two patients with chronic lung diseases. The clinical trial result is highly consistent with our observation on mouse model, and making it a solid basis for future large-scale clinical study.

## RESULTS

### Bronchoscopic isolation of clonogenic airway basal cells

**Figure 1:**
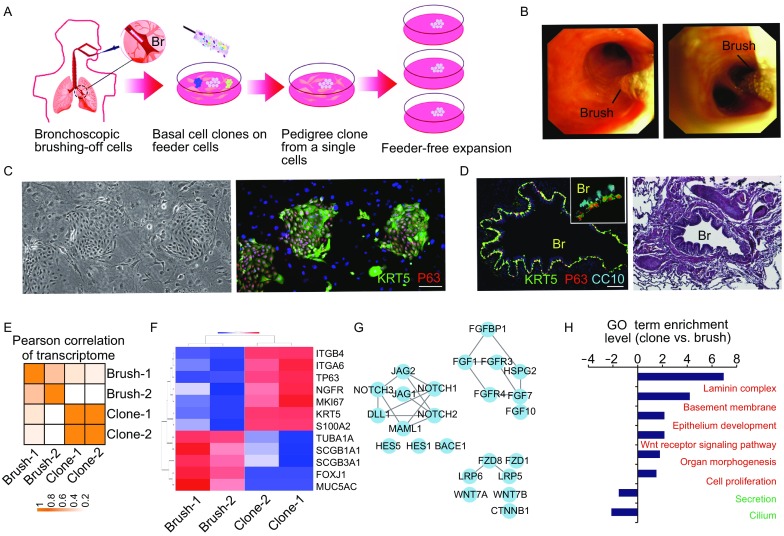
**Isolation and characterization of BCs from SOX9**^**+**^ **human airway**. (A) Diagram showing the process of clonogenic BCs isolation and expansion. (B) Bronchoscopic image showing brushing of cells from human airway. (C) Left, BC colonies grown on feeder cells; right, anti-KRT5 and anti-P63 immunostaining of BC colonies with nuclei counterstain. Human sample number *n* = 10. Scale bar, 100 μm. (D) Left, BCs in human airway by anti-KRT5 and anti-P63 immunostaining. Inset, high magnification with club cell (CC10+, cyan color) costaining; right, hematoxylin & eosin staining of the same section. Br, bronchus. Scale bar, 100 μm. (E) Heatmap showing transcriptome profile correlation value of BC clones and brush-off tissues. (F) Expression heatmap of selected, differentially expressed genes (*P* < 0.05) comparing BC clones and brush-off tissues. (G) Protein-protein interaction network of selected genes with high expression level in BC clones. (H) Enriched gene ontology classes of BC clones versus brush-off tissues

In current study, we worked on the P63^+^/KRT5^+^ BCs in the airway epithelium of human lung which could possibly be the counterpart of mouse DASC. The workflow of BC isolation and expansion is summarized in Fig. 1A. Approximately 20,000–30,000 cells were brushed off from the luminal surface of donor’s 3rd–4th order bronchus using a 2-mm bronchoscopic brush (Wimberley et al., 1982) (Fig. 1B). The brushed-off cells were seeded onto embryo-derived feeder cells with the culture medium favoring BC growth (Zuo et al., 2015; Wang et al., 2015). After seeding 5,000 live cells onto 6-well plate, 9 (±2) cells grew up into visible tight colonies 3–5 days later with expression of human nucleus specific antigens, lung progenitor marker NKX2.1 and proliferation marker KI67 (Figs. 1C and S1A). All of the P0 colonies were confirmed epithelium origin (E-cadherin^+^, Fig. S1A) and stained double positive for airway basal cell markers KRT5 and P63 (Fig. 1C and 1D). We did not observe any P63 single positive colonies (Vaughan et al., 2015). Considering that BCs take for about 20% of total cell number in brushed samples of 3rd–4th order bronchus, it appeared that approximately 1% of the BCs in human airway could be clonogenic lung epithelium progenitors.

We seeded one single BC onto feeder cells and grew them into one single colony which was then picked up by cloning cylinders and passaged continually. The latest passage of BC clones had gone through 50 doublings (=10^15^ fold expansion) in our lab. The single cell-derived BC clones and their original brush-off tissue samples were analyzed by high-throughput RNA sequencing (RNA-Seq). On average, we detected 16,230 genes and 25,223 transcripts. Thus, more than 60% of known human genes and transcripts were expressed in clonogenic BCs. Gene expression value correlation analysis showed that the clone transcriptome profiles are distinct from their original brush-off tissues, but the two clones from two independent persons share very similar transcriptome (Fig. 1E, Pearson correlation coefficient = 0.95). Single nucleotide polymorphism (SNP) analysis showed that BC clones have around 70% less polymorphism comparing to the brush-off tissues, which is in consistency with their single cell origination. High expression of BC markers (KRT5, P63, NGFR and S100A) and another putative mouse stem cell marker integrin α6β4 (Chapman et al., 2011) were observed in clones. In contrast, clonogenic BCs do not express other bronchial or alveolar lineage markers as shown by RNA-Seq and confirmed by immunostaining (Figs. 1F and S1B). Protein-protein interaction analysis of overexpressed genes indicated three major signal molecule networks including Notch1/2/3, FGF10/7 and Wnt7 ligand and their downstream components. All three signaling networks are previously known to play essential roles in embryonic lung development (Bellusci et al., 1997; Rajagopal et al., 2008; Tsao et al., 2008) (Fig. 1G). Gene ontology (GO) term analysis demonstrated critical biological processes enriched in BCs (Fig. 1H).

### Clonal analysis of SOX9^+^ BCs

**Figure 2:**
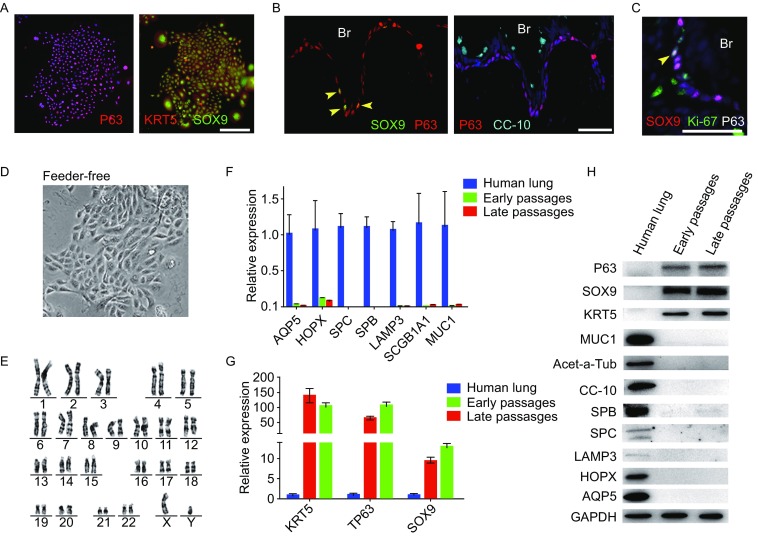
**Feeder-free expansion of SOX9**^**+**^** BCs**. (A) Immunostaining of SOX9^+^ BCs with anti-P63, anti-KRT5 and anti-SOX9 antibodies. (B) SOX9^+^ BCs in rugae of 3rd order human airway by anti-SOX9, anti-P63 and anti-CC10 immunostaining. Scale bar, 100 μm. (C) SOX9^+^ BCs in rugae of 3rd order human airway by anti-KI67 immunostaining. (D) BC colony cultured on feeder-free condition. (E) Karyotyping of cultured BCs. (F) qPCR showing alveolar and bronchial epithelium marker gene expression of human lung sample and SOX9^+^ BCs in early (P2) and late (P8) passages. *n* = 3, biological replicates. Error bars, S.E.M. (G) qPCR showing progenitor cell marker (Krt5, P63 and SOX9) gene expression of human lung sample and SOX9^+^ BCs in early (P2) and late (P8) passages. *n* = 3, biological replicates. Error bars, S.E.M. (H) Western blotting showing marker gene expression of human lung sample and SOX9^+^ BCs in early (P2) and late (P8) passages

Importantly, RNA-Seq data also showed that clonogenic BCs highly express SOX9 (Sex Determing Region Y- Box 9), a transcriptional factor known to be enriched in branching tips of developmental lung. In embryonic development, SOX9 activity is required to maintain the undifferentiated status of distal lung progenitor and disruption of SOX9 function prevents adult alveoli formation (Perl et al., 2005; Rockich et al., 2013). Here we confirmed SOX9 expression in P63^+^/KRT5^+^ BC clones by immunostaining (Fig. 2A). Accordingly, by histological examination of human 2nd order (Fig. S2) and 3rd–4th order airway (Fig. 2B), we observed 1.3% ± 0.3% and 1.7% ± 0.5% SOX9-expressing P63^+^ BCs, respectively. The proportion of SOX9^+^ cells in total BCs is very close to our estimation in clonogenic assay as mentioned above (~1%), suggesting SOX9 as a marker to distinguish clonogenic BCs vs. other non-clonogenic BCs. Interestingly, we noticed that there are a few invaginations (rugaes) in 2–4 order human airway epithelium and the SOX9^+^ BCs are exclusively located near the base of the rugaes. There are averagely 3 (±1) SOX9^+^ BCs in each individual rugae. Further immunofluorescent examination showed a very small portion of them (<1%) are proliferative (KI67^+^) (Fig. 2C). Of note, SOX9^+^ BCs can also be isolated and expanded from those small airway (~1 mm diameter) samples, which is accessible by open-chest surgery or autopsy but not by bronchoscopy (data not shown).

The whole brushing sampling and SOX9^+^ BCs cloning procedure was carried out on 15 individuals with a recovery rate of 100%. Donors are from 4 different disease categories including 5 normal healthy volunteers, 2 bronchiectasis patients, 3 chronic COPD patients, and 5 interstitial lung disease (ILD) patients with pulmonary fibrosis. The SOX9^+^ BCs from different categories of diseases showed no apparent difference in colony morphology (Fig. S3A) or marker expression (Fig. S3B). Their clonogenic efficiency seemed similar—but still need future investigation in much larger cohort to get statistically meaningful conclusion.

We further analyzed SOX9^+^ BCs at single cell resolution. 5 single cells from one person in normal group were selected at Passage 0 and expanded to Passage 1 and Passage 2. Great variation of their clonogenic capacity was observed at Passage 1 (coefficient variation = 59.9%) and Passage 2 (coefficient variation = 75.7%). Similar clonogenicity variation was observed in individuals from other disease categories and the average coefficient variation of all clones is 52.2% (Fig. S3C).

SOX9^+^ BCs grown on feeder cells can be transferred onto petri dish pre-coated with collagen fibers for feeder-free culture. The feeder-free cultured SOX9^+^ BC can also form colonies though their cell-cell contact within one colony is less tight comparing to those on feeders (Fig. 2D). The feeder-free cultured BCs are able to be passaged for at least 30 doublings with no obvious morphology change. Karyotyping indicated their stable genetic characteristics along with passaging (Fig. 2E). Quantitative analysis of progenitor markers (KRT5, P63 and SOX9) and lung epithelium lineage markers at both RNA and protein level indicated that there is no spontaneous differentiation of BCs in the culture process (Fig. 2F–H).

### Xeno-transplanted SOX9^+^ BCs give rise to human lung *in vivo*

**Figure 3:**
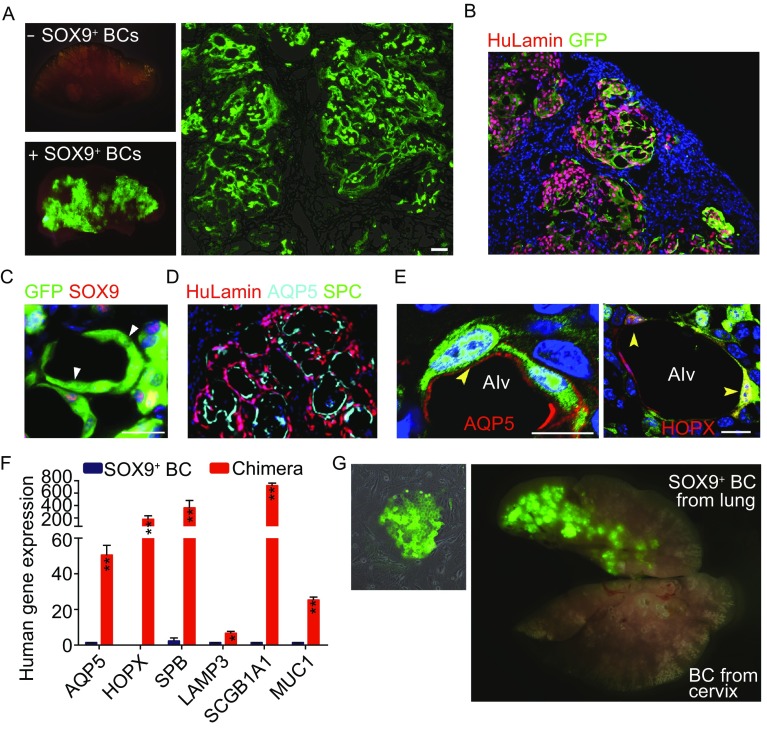
**Transplantated SOX9**^**+**^** BCs regenerate functional human lung** ***in vivo***. (A) Left, direct fluorescence image under stereomicroscope showing NOD-SCID mouse lung without (upper panel) or with (lower panel) GFP-labeled SOX9^+^ BC transplantation. Right, cryo-section and direct fluorescence imaging of transplanted GFP-labeled SOX9^+^ BCs in lung parenchyma. Scale bar, 100 μm. (B) Immunofluorescence imaging of transplanted GFP-labeled SOX9^+^ BCs in lung parenchyma with human specific Lamin A+C marker costaining. (C) Fully differentiated GFP^+^ cells lost SOX9 marker expression (arrowhead indicated). Scale bar, 10 μm. (D) Confocal image with human specific Lamin A+C immunostaining (HuLamin) showing regenerated type I (AQP5^+^) alveolar cells. No type II (SPC^+^) cells were observed. (E) Confocal image showing regenerated AEC1 (AQP5^+^ and HOPX^+^). AQP5 as a membrane-bound protein distributes on surface of GFP^+^ cells. Arrowheads indicated the overlay of HOPX with GFP signal in nucleus. Scale bar, 20 μm. (F) qPCR with human specific primers showing alveolar and bronchiolar epithelium marker gene expression in SOX9^+^ BC transplanted chimeric lung (AEC1: AQP5 and HOPX; AEC2: SPB and LAMP3; bronchiolar cells: SCGB1A1 and MUC1). Biological replicates, *n* = 3. Error bars, S.E.M. (G) Left, clonogenic BCs isolated from human cervix epithelium obtained by biopsy. Right, transplantation of equal numbers of BCs from lung and cervix indicated different incorporation efficiency

Next we examined whether the SOX9^+^ BC could differentiate and regenerate lung tissue by transplanting such cells into mouse lung parenchyma. Firstly, immunodeficient NOD-SCID mice were subjected to bleomycin intratracheal instillation, which lead to rapid onset (8 days after bleomycin) damage of centrilobular and surrounding regions as shown by microCT-scan and immunostaining. Masson trichrome staining for collagen and α-SMA immunostaining indicated severe tissue fibrosis of mouse lung at later time points (Zhang et al., 1996) (Fig. S5A–C). Scarce endogenous mouse p63^+^/Krt5^+^ distal airway stem cell expansion was observed in damaged lung parenchyma as reported previously (Vaughan et al., 2015) (Fig. S5D). Then we intratracheally delivered (Zuo et al., 2015) 1 × 10^6^ GFP-labeled SOX9^+^ BCs into the injured mouse lung and analyzed the lung 3 weeks after transplantation. As shown in Fig. 3A, we observed large-scale incorporation of GFP^+^ human SOX9^+^ progenitors and their progeny into mouse lung. Direct fluorescence after tissue sectioning showed distribution of GFP^+^ human cells in mouse distal lung, some of them are morphologically indistinguishable from neighboring GFP^−^ mouse lung structures (Fig. 3A). The chimerism of human-mouse lung was further confirmed by human-specific nucleus antigen Lamin A+C co-staining with GFP (Fig. 3B) and qPCR with human specific GAPDH primers (Fig. S6A). A few fully differentiated human cells have lost SOX9 marker expression and form air-sacs of similar size to mouse alveoli with AEC1 marker (AQP5 and HOPX) expression (Fig. 3C–E). Some transplanted GFP^+^ human cells could also incorporate into bronchiolar region of lung, where some of them gave rise to Club cell with CC10 marker expression while a few others became ciliated cells (acetylated-tubulin^+^, FOXJ1^+^), respectively (Fig. S6B–D). However, we hardly observed human SPC^+^ AEC2 in transplanted mouse lung. The differentiation potential of SOX9^+^ BCs was further confirmed by qPCR analysis of multiple marker genes with human specific primers. Both AEC1 and bronchiolar cell marker genes were strongly expressed in the chimera. For AEC2 marker genes, though SPB and LAMP3 were highly expressed, we did not detect SPC expression in the chimera, which was consistent with the immunostaining result (Fig. 3F).

In control experiment, we found that the transplanted SOX9^+^ progenitors cannot incorporate into non-injured healthy mouse lung or porcine pancreatic elastase-injured mouse lung (data not shown). Also, human lung-derived fibroblast cells (data not shown) or human cervix-derived P63^+^/KRT5^+^/SOX9^+^ progenitor cells (Figs. 3G and S6E) can barely incorporate into injured mouse lung either. This data indicated the tissue specificity of different adult stem/progenitor cells.

### Regenerated lung by SOX9^+^ BC transplantation contributed to mouse pulmonary function

**Figure 4:**
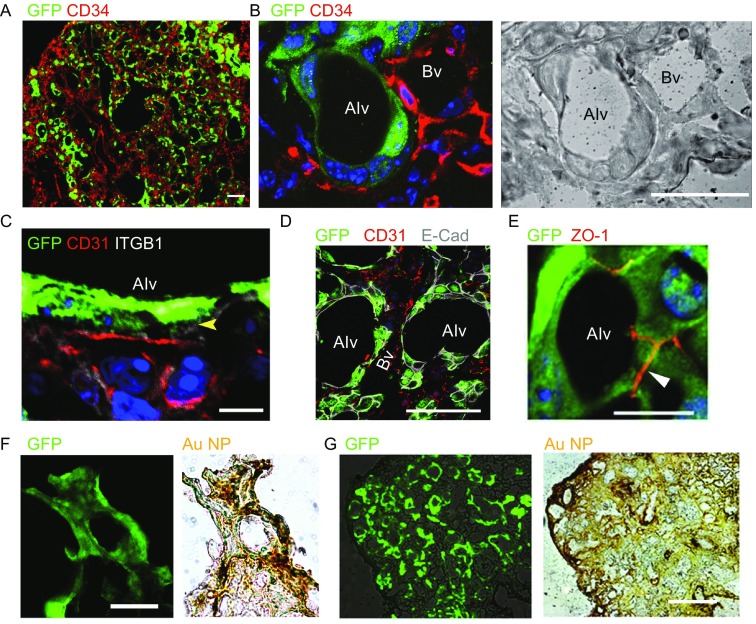
**Regenerated alveoli with functional epithelium-capillary system**. (A) Transplanted SOX9^+^ BCs (anti-GFP) and capillary endothelium marker (anti-CD34). Scale bar, 100 μm. (B) Confocal image of SOX9^+^ BCs regenerated alveoli (Alv) and the neighboring capillary blood vessel (Bv). Left, immunofluorescence; right, bright field. Scale bar, 20 μm. (C) Confocal image showing the basement membrane (ITGB1^+^, white color, arrowhead indicated) between regenerated alveoli epithelium and capillary endothelium (CD31^+^). Scale bar, 10 μm. (D) Confocal image showing the cell adherens junction (E-cadherin^+^, white color) between regenerated alveoli epithelial cells. Scale bar, 20 μm. (E) Confocal image showing the cell tight junction (ZO-1^+^) between regenerated alveoli epithelial cells. Scale bar, 20 μm. (F) Direct fluorescence image of the transplanted GFP-labeled SOX9^+^ BCs (green) and bright-field image of tail vein delivered gold nanoparticles (AuNPs) of the same region (brown). Scale bar, 100 μm. (G) Direct fluorescence image of the transplanted GFP-labeled SOX9^+^ BCs (green) and bright-field image of intratracheally delivered gold nanoparticles (AuNPs) of the same region (brown). Scale bar, 100 μm

Functional alveolar unit requires close epithelium-capillary interaction for exchange of gas, energy and other substances. In the optically cleared mouse lung, we observed branching major blood vessels in transplanted mouse lung (Fig. S7A). We also found that the thin, long-shape human AEC1 aligned together with microvascular vessels which are positive for capillary endothelial markers CD34 and PECAM/CD31, with approximately 1 μm-thick integrinβ-1^+^ basement membrane between epithelium and capillary endothelium (Fig. 4A–C). And engrafted GFP^+^ human cells form adherens junctions and tight junctions with neighboring alveolar epithelial cells as shown by E-cadherin and ZO-1 staining on the border (Fig. 4D and 4E), which makes a closed space to maintain air pressure. In order to examine whether such blood-gas exchanging units are functionally connected with circulation, we developed a gold nanoparticle (AuNP) (Cheng et al., 2008)-based approach to mimic gas exchange and transport *in vivo*. The nanoparticles can be transported in blood and diffuse across cells (like O_2_ and CO_2_) due to its small size (~5 nm), water solubility and lipophilicity, and meanwhile can be detected by histology. One hour after injection of AuNPs into mouse tail vein, we detected significant gold signal in healthy mouse alveoli (Fig. S7B) as well as in GFP^+^ human alveoli (Fig. 4F), indicating the regenerated human tissues are functionally linked with circulation system. On the other side, after intratracheally aspiration of AuNPs, some GFP^+^ part of mouse lung showed significant gold signal, indicating the regenerated human tissues are anatomically linked with atmospheric air (Figs. 4G and S7C). As control, no or very little AuNPs signal was observed in damaged alveolar area by either way of particle delivery (Fig. S7B and S7C). These evidences implicated that the regenerated lung tissue has vascularized gas-exchange capacity, probably through recruitment of self-organizing capillary endothelial cells by SOX9^+^ BCs.

**Figure 5:**
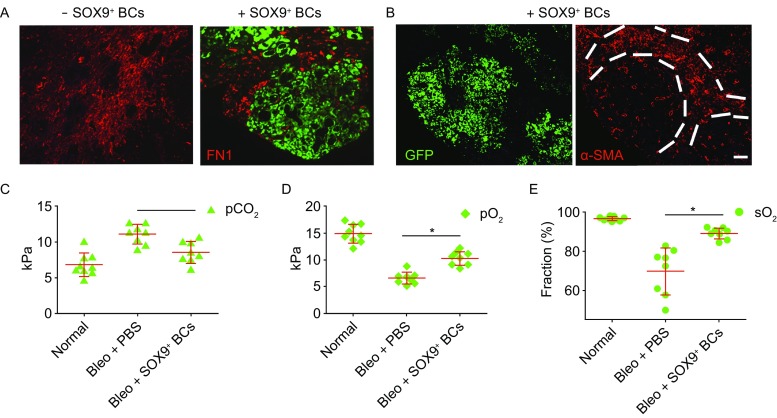
**BC transplantation rescued mouse pulmonary function**. (A) Injured mouse lung without or with GFP-labeled SOX9^+^ BCs transplantation by anti-GFP and anti-Fibronectin co-staining. Scale bar, 200 μm. (B) Left, immunofluorescence image of injured mouse lung transplanted with GFP-labeled SOX9^+^ BCs; right, immunostaining on the same section showing exclusion of α-SMA+ myofibroblasts from GFP^+^ area. Scale bar, 200 μm. (C) CO_2_ partial pressure of mouse arterial blood before and 1 month after bleomycin-induced injury with or without SOX9^+^ BCs transplantation. Each dot indicates an individual mouse. (D) O_2_ partial pressure of mouse arterial blood 1 month after bleomycin-induced injury with or without SOX9^+^ BCs transplantation. Each dot indicates an individual mouse. (E) O_2_ saturation of mouse arterial blood before and 1 month after bleomycin-induced injury with or without SOX9^+^ BCs transplantation. Each dot indicates an individual mouse

We also found that SOX9^+^ BC transplantation effectively blocked the progression of mouse pulmonary fibrosis manifested as fibronectin accumulation and α-SMA positive myofibroblast expansion (Phan, 2012) in the human cell-enriched area (Fig. 5A and 5B), suggesting that regenerated human lung can replace damaged tissue in mouse model. Accordingly, alveoli regeneration by SOX9^+^ BC transplantation also improved the recipient mouse pulmonary function as shown by the decrease of CO_2_ partial pressure, increase of O_2_ partial pressure and O_2_ saturation in artery blood (Fig. 5C–E).

### TGF-β signaling modulates SOX9^+^ BC proliferation

**Figure 6:**
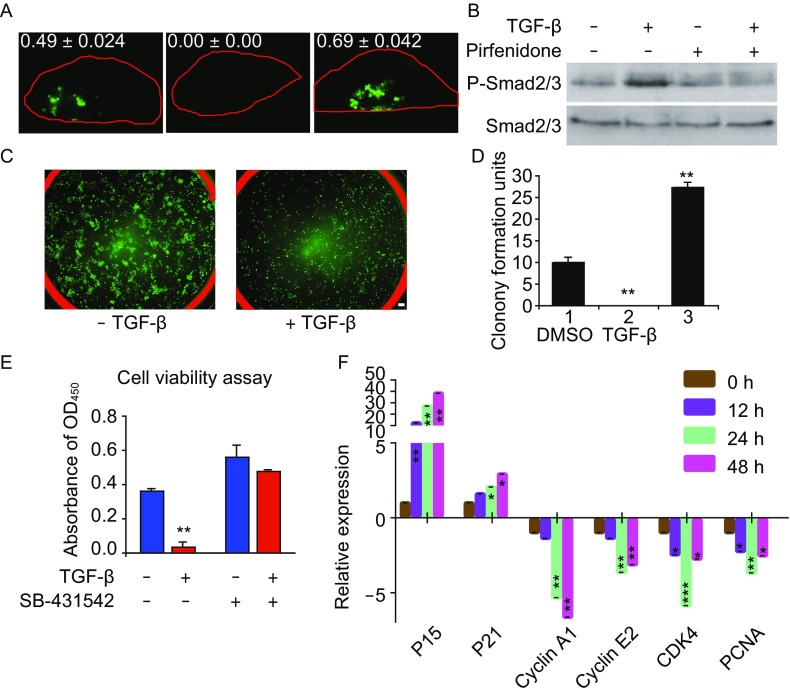
**TGF-β signaling modulates SOX9**^**+**^ **BC proliferation**. (A) Direct fluorescence image of mouse lung transplanted with 1 × 10^6^ GFP-labeled SOX9^+^ BCs under dissection microscope. Each lung was from mouse with indicated treatment and harvested 7 days after transplantation. The left lobes were analyzed and the GFP^+^ cell numbers (×10^6^) were counted by flow cytometry analysis. Biological replicates, *n* = 3. PFD, Pirfenidone. (B) SOX9^+^ BCs were stimulated with 10 ng/mL TGF-β for 2 h, with or without 1 mg/mL Pirfenidone treatment overnight. Western blotting of cell lysates with anti-phosphated-Smad2/3 and anti-total Smad2/3 antibodies was performed to examine the activation of TGF-β pathway. (C) Direct fluorescence imaging of GFP-labeled SOX9^+^ BCs cultured in a 6-well plate in the absence or presence of 10 ng/mL TGF-β. Scale bar, 200 μm. (D) Quantification of clonogenicity of SOX9^+^ BCs in the presence of 10 ng/mL TGF-β or 10 mmol SB. SB, TGF-β type I receptor inhibitor SB-431542. Technical replicates *n* = 3. (E) WST viability assay of SOX9^+^ BCs treated by 10 ng/mL TGF-β or 5 mmol TGF-β inhibitor SB-431542, or their combination. Technical replicates *n* = 3. (F) qPCR showing cell cycle-related gene expression level of SOX9^+^ BCs with 10 ng/mL TGF-β treatment for indicated h. Biological replicates, *n* = 3

To further improve the transplantation efficiency of SOX9^+^ BCs, we screened multiple drugs and found Pirfenidone, an FDA approved anti-pulmonary fibrosis drug (King et al., 2014) could facilitate the SOX9^+^ BC transplantation efficiency significantly. Interestingly, transforming growth factor-β (TGF-β) had the opposite effect (Figs. 6A and S8A). This discovery prompted us to study the underlying molecular and cellular mechanism. We found that Pirfenidone treatment can abolish TGF-β-induced phosphorylation of SMAD2/SMAD3 (Fig. 6B). In turn, TGF-β treatment significantly suppressed the clonogenicity and cell viability of SOX9^+^ BCs, which can be rescued by the SMAD2/SMAD3 inhibitor SB-431542 (Fig. 6C–E). Simutaneously, the expression of p15(INK4B), a G_1_ cell cycle inhibitor, was strongly induced by TGF-β treatment together with mild change of some other cell cycle-related genes (Fig. 6F). TGF-β had little effect on the apoptosis of SOX9^+^ BCs (Fig. S8B). Collectively these experiments showed that the TGF-β/SMAD/P15 signaling axis could effectively modulate SOX9^+^ BC proliferation. Similar proliferation inhibitory effect of TGF-β/SMAD was recently reported on TBC as well (Mou et al., 2016).

### Autologous SOX9^+^ BCs transplantation clinical trial in bronchiectasis patients

Bronchiectasis is a chronic lung disease radiographically characterized by permanent pathologic dilation of the small and medium-sized bronchi, which may lead to respiratory failure and eventually to death. Patients with bronchiectasis, if left untreated, will have a continual decrease of their pulmonary function. Current pharmacological strategies to treat bronchiectasis such as antibiotics, mucolytics and anti-inflammatory agents could only control the disease exacerbation but not improve the pulmonary function nor repair the damaged lung tissue (ten Hacken et al., 2007). To explore the clinical feasibility of autologous SOX9^+^ BC transplantation, we conducted a pilot trial aiming to treat bronchiectasis by regenerating functional human lung. The general trial protocol and the cell manufacturer (Regend Therapeutics Co.Ltd) were archived by China Food and Drug Administration (CFDA) and National Health and Family Planning Commission of China, and the trial was performed in national approved stem cell clinical research institute (Southwest Hospital) after strict ethic commission review of preclinical data (A part of but not all preclinical data was released in the current manuscript).

Two patients diagnosed as non-CF bronchiectasis were firstly enrolled for autologous SOX9^+^ BC transplantation on April, 2016. Both patients are men in 50s, non-smokers. Patient 1 was diagnosed as bronchiectasis 8 years ago with productive cough and dyspnea on exertion symptom, which worsens continually under regular pharmacological treatment. CT scan shows multiple bronchial cylinder dilation and patchy consolidation in his lung. Patient 2 was diagnosed as bronchiectasis and COPD decades ago, with productive cough and dyspnea on exertion symptom, which worsens continually under regular pharmacological treatment. CT scan shows multiple bronchial cystic dilation, thicken bronchial wall and patchy consolidation in his lung.

**Figure 7:**
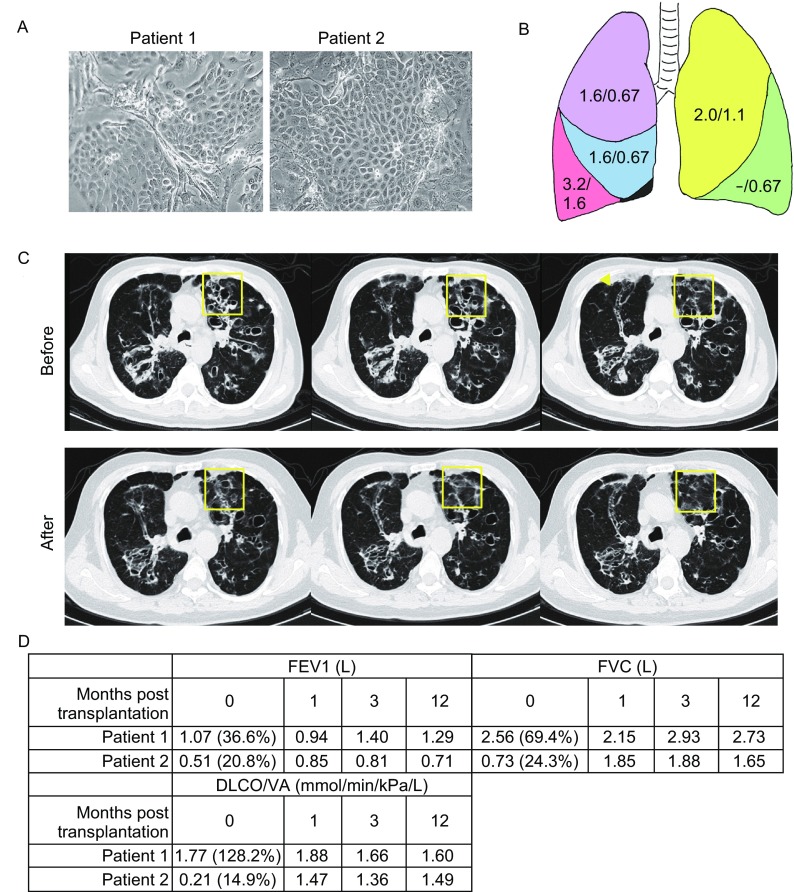
**Autologous SOX9**^**+**^** BC transplantation: a pilot clinical study**. (A) Cultured SOX9^+^ BCs from two patients of bronchiectasis. (B) Diagram showing the number of SOX9^+^ BCs (×10^7^) transplanted into each lobe of Patient 1/Patient 2. Five lobes are labeled with different colors. Note the Patient 1 undertook left lower lobectomy a decade ago. (C) Representative images of consecutive CT scan show the regional recovery of bronchiectasis (yellow square) 1 year after autologous SOX9^+^ BC transplantation in Patient 2. (D) Measurement of pulmonary function and exercise capacity in both patients: FEV1 (forced expiratory volume in 1 second), FVC (forced vital capacity), DLCO/VA (diffusing capacity of the lung for carbon monoxide adjusted by the alveoli volume) were shown as absolute value and percentage to predicted level. For each parameter, percentage >80% was regarded as clinically normal

For both patients, tissues were bronchoscopically collected from random region of left upper lobe and right upper lobe and transported to GMP (Good Manufacture Practices) level tissue culture facility for SOX9^+^ BC isolation and expansion (Fig. 7A). Isolated SOX9^+^ BCs were cultured on clinical-level feeder cells and then shifted to feeder-free culture condition. Totally 1 × 10^6^/kg body weight of SOX9^+^ BCs were infused into distinct lobes of patients through bronchoscopy (Tzouvelekis et al., 2013) (Fig. 7B). Clinical status of patients was evaluated 1 day before and 1, 3 and 12 months after cell transplantation. Although it is almost impossible to directly track unlabelled transplanted cells in human, we did observe regional repair of cystic dilation after cell transplantation by high-resolution computed tomography (HRCT) scan for Patient 2 (Fig. 7C). The thickened bronchial wall also became thinner after cell therapy for Patient 2. Spirometry results indicated remarkable recovery of pulmonary function in both patients after transplantation as measured by FEV1, FVC and DLCO/VA (Fig. 7D). Importantly, no aberrant cell growth or other related adverse events were observed during the whole follow-up time. In the last follow-up (20 months after transplantation), Patient 1 described improvement of dyspnea, improvement of exercise capacity, less productive cough and less times of exacerbation after cell therapy; Patient 2 described less productive cough and less times of exacerbation after cell therapy. As it is well documented that bronchiectasis is a permanent, irreversible disease that cannot resolve spontaneously or with regular medicine, the recovery of patients suggested high probability that transplanted SOX9^+^ BCs were able to regenerate functional lung in human, which is consistent with our observation in animal models. And we will continue life-long observation on the two patients.

## DISCUSSION

In the current study, we revealed that a small population of SOX9^+^ BCs in adult airway can regenerate human lung epithelium. A few SOX9^+^ BCs brushed off from human airways can be expanded to sufficient number in feeder-free condition and transplanted into injured mouse lung to regenerate human air exchanging units. In unperturbed lung, the SOX9^+^ BCs are located in the base of airway epithelium invagination, which is reminiscent of intestinal stem cells residing in gut crypt compartments. However unlike the intestinal stem cells which are constantly in active cell cycle to replenish gut epithelium in a fast turn-over mode (Clevers, 2013), the SOX9^+^ BCs in airway are quiescent most of the time, and could only be activated once a particular injury signal from more distal lung was received. Like its counterpart p63^+^/Krt5^+^ BCs in mouse, endogenous SOX9^+^ BCs might migrate towards inflamed parenchymal region and rebuild the respiratory tree by proliferation and differentiation. We and previous studies found that there are P63^+^ and/or KRT5^+^ cells enriched in the damaged alveolar region of many lung disease patients (including those with COPD, pulmonary fibrosis and bronchiectasis) (Chilosi et al., 2002; Asano et al., 2011; Smirnova et al., 2016), which could be the progeny of endogenous SOX9^+^ BCs in the middle of differentiation process. Interestingly there was almost no SPC^+^ AEC2 generated in our xeno-transplantation model, which suggested the limitation of the SOX9^+^ progenitor potency and also the possibility that the traditional concept of human AEC2 as the progenitor of AEC1 may not be correct in this circumstance.

The pattern that SOX9^+^ BCs adopt to regenerate the respiratory tree resembles the natural development process of lung in gestation, which raises an open question as whether SOX9^+^ BCs are the remains of previously documented SOX9^+^ progenitors in embryonic lung. In embryonic lung, SOX9^+^ embryonic progenitors are enriched in distal bud tips of respiratory tree at canalicular stage (Perl et al., 2005). Interestingly, previous report demonstrated that intravenous transplantation of human canalicular-stage embryonic lung cell mixture into NOD-SCID mice can give rise to chimeric lung (Rosen et al., 2015). Similarly, a more recent report showed mouse SOX9^+^ progenitors in embryonic lung can be grown *in vitro* as organoid and transplanted to generate mouse alveoli (Nichane et al., 2017). Altogether these work supported the concept that SOX9^+^ cells are lung progenitors in both embryonic and adult lung.

One important technical advance we bring out in this work is the system to selectively expand SOX9^+^ BCs in a feeder-free condition. Previously through bronchoscopic brushing followed by routine basal cell culture, Crystal et al., obtained P63^+^/KRT5^+^/SOX7/15/4^+^ basal cells but not the rare SOX9^+^ subpopulation (Hackett et al., 2011) . Here from as few as one single SOX9^+^ BC, we can expand it to 5 × 10^7^ purely undifferentiated cells within 3–4 weeks. Therefore we can acquire a homogeneous population of regenerative cells with uniform characteristics, which is crucial for cell quality control in further clinical application.

Most importantly, we showed that SOX9^+^ BCs cultured under GMP guidelines can be applied clinically in order to reconstitute human lung for devastating chronic lung disease treatment. To the best of our knowledge, this is the first successful attempt to regenerate human large inner organ based on cell replacement strategy. As demonstrated in hematopoietic, skin and corneal regeneration field, autologous stem/progenitor cell transplantation strategy has been successfully applied to treat multiple devastating diseases. We show here that supplement of expandable SOX9^+^ BCs by autologous transplantation could repair the damaged lung in two bronchiectasis patients in both pulmonary structure and function. Generally the bronchiectasis patients without medical intervention will deteriorate over time, thus the recovery of lung function and structure after cell therapy suggested the efficacy of this strategy, and established a basis for future trials. As the SOX9^+^ BCs are derived from patients’ own airway, it is a recapitulation and augmentation of naturally occurring lung repair process. However after all, our pilot clinical trial is a very preliminary exploratory study so the safety and efficacy of the current strategy still need additional verification in a much larger cohort. Although we have never observed aberrant growth of SOX9^+^ BCs in NOD-SCID mouse model, longer time follow-up on the patients is still necessary to fully eliminate the tumorigeneic possibility. We have demonstrated the differentiation potential of SOX9^+^ BCs in mouse model, but the exact fate of transplanted SOX9^+^ BCs in human lung remains to be proven with future development of non-invasive cell tracking techniques. Furthermore, we have demonstrated that SOX9^+^ BCs derived from normal or diseased people can both give rise to multiple lineages of lung epithelial cells, but detailed quantitative comparisons of the regenerative capacity between normal and diseased persons would require a larger sample size investigation in future.

In conclusion, our study clearly shows the capability of SOX9^+^ BCs to regenerate human lung, proves the concept of chronic lung disease treatment by SOX9^+^ BC transplantation and provides exciting translational opportunities in near future.

## METHODS

### Human tissue collection

Patients without or with chronic lung diseases (COPD, bronchiectasis and ILD) were diagnosed by ATS/ERS criteria. All individuals went through thorough medical examination before sampling. The bronchoscopic procedure for sampling was performed by board-certified respiratory physicians using a flexible fiber-optic bronchoscope (Olympus, Japan). Before the bronchoscopy, oropharyngeal and laryngeal anesthesia was obtained by administration of 2 mL of nebulized 4% lidocaine, followed by 1 mL of 2% topical lidocaine sprayed into the patient’s oral and nasal cavities. After the bronchoscope was advanced through the vocal cords, 2 mL of 2% lidocaine solution was instilled into the trachea and both main bronchi through the working channel of the bronchoscope. Then a disposable 2-mm brush was advanced through the working channel of the fiberoptic bronchoscope and used to collect airway epithelial cells by gently gliding the brush back and forth 1 or 2 time in random regions of trachea or 3–4 order bronchi in the right or left lobe. No obvious differences were observed between the BC clones isolated from 3rd vs. 4th order bronchi, or from different lung lobes. For bulk lung sampling, normal human lung bulk samples were collected from unaffected lung area of lung cancer patients with open chest surgery. All the human tissues were obtained following clinical SOP under patient’s consent and approved by Southwest Hospital Ethics Committee (Chongqing, China) and Shanghai East Hospital Ethics Committee (Shanghai, China).

### Isolation and culture of human SOX9^+^ BCs

To isolate the SOX9^+^ BCs, 2 mm brush with samples were cut with scissors into 1 cm pieces. After removing sputum, the brush pieces were directly digested with dissociation buffer including DMEM/F12 (Gibco, USA), 2 mg/mL protease XIV (Sigma, USA), 0.01% trypsin (Gibco, USA) and 10 ng/mL DNase I (Sigma, USA). Specimens were incubated at 37°C for an hour with gentle rocking. Alternatively, human small airway were dissected from a bulk of lung tissue and digested in the same dissociation buffer at 37°C overnight. Dissociated cells were passed through 70-μm Nylon mesh (Falcon, USA) to remove aggregates and then washed twice with cold F12 medium. Cell viability was assessed by exclusion of trypan blue dye. Cell pellets were collected by centrifuge of 200 ×*g* and plated onto mitomycin-inactivated 3T3 feeder cells in BC culture medium for lung (BCM-L) including DMEM/F12 (Gibco, USA), 10% FBS (Hyclone, Australia), antibiotics, amphotericin and growth factor cocktail as previously described (Zuo et al., 2015). Under 7.5% CO_2_ culture condition, the SOX9^+^ BC colonies emerged 3–5 days after plating, and were digested by 0.25% trypsin-EDTA (Gibco, USA) for 3–5 min for passaging. Typically, SOX9^+^ BCs are passaged every 5 to 7 days and split at 1:7 ratio. To obtain single cell-derived clone, cells are digested into single cells, loaded through 40-μm Nylon mesh and seeded with extremely low density, then a single colony grown up from a single cell was picked up by clone cylinder (Sigma, USA) and high vacuum grease after its neighboring colonies were cleared by scraper to ensure the pedigree purity. For feeder-free culture of SOX9^+^ BCs, feeder cells were removed by differential trypsinization with 0.05% trypsin (Gibco, USA) and SOX9^+^ BCs were plated with high density onto dishes pre-coated with 15% cold collagen type I (Corning, USA) and 20% Matrigel (Corning, USA) for further expansion.

For labeling of cells by GFP, pLenti-CMV-EGFP plasmid was transfected into 293T cells together with lentivral packaging mix (Life Technologies, USA). Lentivirus supernatant produced by 293T was collected, filtered and cryo-preserved before use. To infect SOX9^+^ BCs, 0.5 mL lentivirus containing medium was directly added to 2 mL cell culture medium with 10 μg/mL polybrene and incubated for 12 h. The overall labeling efficiency of cells is above 95%. To confirm that GFP containing virus will not spread between cells after labeling, we co-cultured GFP-labeled SOX9^+^ BCs with mCherry-labeled (pLenti-CMV-mCherry) SOX9^+^ BCs for 5 days and did not observe any yellow color cells.

### Immunofluorescence staining

For immunofluorescence staining, cells were fixed by 3.7% formaldehyde, and then incubated with 0.3% Triton X-100 to improving the cell permeability for 10 min. Paraffin- or cryo-embedded tissues were sectioned and subjected to antigen retrieval in citrate buffer (pH 6.0, Sigma, USA) in microwave oven for 20 min before staining. 10% normal donkey serum (Jackson ImmunoResearch) was used to block the non-specific antigen. Primary antibodies used in this work include BC markers:KRT5 (1:200, EP1601Y, Thermo), P63 (deltaN, 1:200, 4A4, Abcam), E-cadherin (1:200, H-108, Santa cruz), SOX9 (1:200, ERP14335-78, Abcam), SOX9 (1:200, AF3075, R&D); AEC markers: AQP5 (1:1000, EPR3747, Abcam), HOPX (1:500, E-1, Santa Cruz), PDPN (1:500, FL-162, Santa Cruz), SPC (1:200, M-20, Santa Cruz), SPC (1:200, FL-197, Santa Cruz), LAMP3 (1:200, 12632-1-AP, Proteintech); bronchiolar cell markers: CC10 (1:200, T-18, Santa Cruz), acetylated-α-Tubulin (1:1000, 6-11B-1, Abcam), MUC5AC (1:500, 45M1, Thermo), FOXJ1 (1:200, 2A5, eBioscience); vasculature markers: CD31 (1:100, M-20, Santa Cruz), CD34 (1:1000, EP373Y, Abcam); myofibroblast marker: α-SMA (1:500, 1A4, DAKO), Fibronectin (1:500, F14, Abcam), others: KI67 (1:200, RM-9106, Thermo), GFP (1:200, B-2, Santa Cruz), GFP (1:200, FL, Santa Cruz), GFP (1:200, T-19, Santa Cruz), ITGB1 (1:500, ERP16895, Abcam), Human specific Lamin A+C (1:200, EPR4100, Abcam). Alexa Fluor-conjugated Donkey 488/594/647 (1:200, Life Technologies, USA) were used as secondary antibodies. For antibodies of low reactivity, Biotin-Streptavidin signal amplification system (Life Technologies, USA) was used. After counterstaining with DAPI (Roche, USA), samples were treated with 0.1% Sudan Black (Sigma, USA) for 1 min to remove autofluorescence and then mounted with VECTASHIELD® Mounting medium (Vector labs, USA). Images were visualized under fluorescence microscope (Nikon 80i and Eclipse Ti, Nikon, Japan) or fluorescence stereomicroscope (MVX10, Olympus, Japan). Confocal images were taken under Nikon A1R microscope (Nikon, Japan).

### RNA-sequencing and bioinformatics

SOX9^+^ BCs isolated from two donors and their corresponding brush-off specimens were subjected to RNA-Seq analysis. The total RNA concentration and RIN were measured by Agilent 2100 Bioanalyzer (Agilent). For human SOX9^+^ BCs, 200 ng total RNA sample was purified, and the first-strand cDNA was synthesized using first strand master mix and super script II (Life Technologies). Second strand master mix (Life Technologies) was then used to synthesize the second-strand cDNA. After cDNA purification and adapter ligation, PCR amplification was performed to enrich the cDNA fragments. For brush-off samples, after RNA extraction and quality control, cDNA was prepared using the SMARTer Ultra Low RNA Kit (Clontech) for Illumina sequencing. Low Input Library Prep Kit (Clontech) was then used for library construction. The library quantity and quality was verified by Agilent 2100 Bioanalyzer and real-time quantitative PCR. Then the library is sequenced using Illumina HiSeq 4000. Clean data were acquired from raw data (fastq format) using the NGSQC Toolkit by removing low-quality reads. Clean RNA-seq reads were mapped to the reference genome (Ensembl, GRCh37) using Tophat v2.0.049 using default settings.

With genome mapping result, gene expression level was calculated with RSEM software (v1.2.12). Transcript levels were quantified as fragments per kilobase of transcript per million mapped reads (FPKM). Pearson correlation coefficient between samples was calculated by R scripts (3.2.3). Heatmap was generated using R scripts. The protein-protein interactions were retrieved from Human Protein Reference Database (HPRD, release 9) and visualized with Cytoscape (v3.3.0). SNPs were called by GATK (v3.4-0).

### Karyotyping

To arrest SOX9^+^ BCs in mitosis metaphase, cells of 75% confluence were treated with 1 μg/mL colchicines for 7 h and digested into single cells by 0.25% trypsin. Then the cells were incubated by 0.4% KCl at 37°C for 40 min and fixed by 10 mL fixation solution including methanol and glacial acetic acid (3:1) at room temperature for 30 min. Suspension with chromosomes was dropped and spread on slides. Samples on slides were treated by 0.0005% trypsin for 5 min and stained with 15% Giemsa (Sigma-Aldrich, USA). Banding patterns on chromosome spreads were checked for more than 15 mitotic phases and all of them are normal human cells. All cells used for clinical purpose were subjected to karyotyping in prior to transplantation.

### Quantitative reverse transcription PCR

Total RNA from tissues or cells were isolated using the RNeasy mini kit with DNase digestion according to the manufacturer’s instructions (Qiagen). RNA quality was determined by SimpliNano (GE Healthcare). 1 μg total RNA was reverse-transcribed into cDNA with PrimeScript™1st Strand cDNA synthesis Kit (TaKaRa). The real-time PCR assays were performed on an ABI 7500 real-time PCR system (Applied Biosystems) according to the instructions of SYBR® Premix Ex Taq™II (Tli RNaseH Plus, Takara). qPCR reactions were set as following: 95°C for 2 min, then 40 cycles of 95°C for 10 s, and 60°C for 40 s. Melt curve stage was added after PCR amplification stage. The threshold crossing value (Ct) of each transcript was normalized to reference genes (β-Actin or GAPDH). The relative expression level of each genes was calculated using the 2^−ΔΔCt^ method. Sequence of primer pairs for qPCR was listed in Supplementary Table.

### Animal tissue histology

All animal experiments were conducted according to guidelines approved by University Association for Laboratory Animal Science. NOD-SCID mice (female or male, 6–10 weeks, The Jackson Laboratory, USA) were euthanized at proper time points and the diaphragm was carefully cut open without touching the lung. *In situ* fixation by injecting 3.7% formaldehyde (Sigma, USA) through trachea was performed using 29 G needle. Then the lung was dissected and fixed in 3.7% formaldehyde at 4°C overnight. For cryosection, the fixed lung was settled by 30% sucrose before embedding into the Tissue-Tek O.C.T compound (Sakura, Japan), the 5–10 μm sections were cut using a cryotome (Leica microsystem, Germany). For paraffin section, the lung was dehydrated by gradient ethanol and processed in an automatic tissue processor, then embedded into the paraffin blocks. All the samples were sliced into 5–7 μm thickness using microtome (Leica microsystem, Germany). Haematoxylin and eosin (H&E) staining was performed following standard protocol. Masson trichrome staining was performed following the manual of Trichrome Staining Kit manual.

### Small animal micro-CT

Micro-CT was used to monitor mouse lung damage before transplantation. Mice were anesthetized through intraperitoneal injection with chloral hydrate and fixed with tap. Lung image was obtained using a volumetric micro-CT scanner without respiratory gating (Triumph^TM^, Gamma medica-ideas, Northridge, USA). Scanning was performed at 70 kV, 350 μA. The number of projections were 512 slices and total acquire time was around 4.27 min. All data were converted into digital imaging by TRIUMPH ‘X-O’ CT system software.

### SOX9^+^ BC transplantation in mouse

Adult NOD-SCID mice (female or male, 6–10 weeks, The Jackson Laboratory, USA) maintained in SPF animal facilities were used for xeno-transplantation experiments. Mouse lung was injured by intratracheally instilling with 3 U/kg body weight bleomycin (Selleckchem, USA) eight days prior to transplantation. Mouse lung was monitored by small animal micro-CT before transplantation to verify lung damage. Then mice were anesthetized by I.P injection of 3% chloral hydrate and rested on a stand gesture. One million GFP-labeled cells were suspended in 50 μL PBS and used for transplantation of each mouse. Intratracheal aspiration was performed by injecting the cells into trachea via mouth. Three weeks after transplantation, the lung samples were collected for analysis.

### Fluorescence compatible optical clearing of lung sample

Optical clearing of SOX9^+^ BC transplanted lung was performed following the SeeDB protocol of Meng-Tsen Ke et al., (2013) with minor modification. Briefly, a whole lobe of lung was fixed by 3.7% formaldehyde, and then transferred into 20%, 40%, 60%, 80% and 100% (*w*/*v*) fructose solution containing 0.5% α-thioglycerol. In each gradient solution lung was incubated for 12 h at RT. In the end, lung was transferred into SeeDB solution (80.2% *w*/*w* fructose) for 48 h to 72 h. The GFP fluorescence and blood vessels were directly visualized by fluorescence stereomicroscope.

### Tracing intravascular transport by nanoparticles

Water soluble 5 nm gold nanoparticles (AuNPs) were synthesized as described previously (Cheng et al., 2008) with minor modifications: 0.25 mmol tetra-n-octylammonium bromide (TOAB) and 0.6 mmol dodecylamine (DDA) dissolving in 5 mL of toluene was mixed with 0.53 mmol HAuCl_4_ solution (30% in HCl solution). A 2 mmol cold NaBH4 aqueous solution was added into the organic phase and stirred vigorously for 2 h. The DDA-AuNPs were collected by precipitation in 40 mL of ethanol and then redispersed in 3 mL of chloroform. Next, MeO-PEG-SH (MW = 5000) and the DDA-stabilized Au nanoparticles were mixed in chloroform and stirred overnight. The organic phase was washed twice by water and then evaporated under vacuum. The residues were washed three times by water and purified by centrifugation.

To trace the intravascular transport route into lung, 10 mmol/L AuNPs were dissolved in 50 μL PBS and injected intravenously into mouse tail. To trace the aspiration route into lung, 10 mmol/L AuNPs were dissolved in 50 μL PBS and instilled intratracheally into mouse lung. One hour later, the lung was collected and then briefly fixed in 3.7% formaldehyde for 30 min on ice, and then embedded into the Tissue-Tek O.C.T compound followed by cryosections. GFP signal on tissue slide was captured by direct immunofluorescence and then the same slide was stained with LI Silver Enhancement Kit following manufacturer’s instruction (Thermo, USA). Brown color indicates the precipitation of AuNPs after reaction with silver.

### Western Blotting

Cells were washed in cold PBS and harvested by plastic scraper. Collected lung tissues were washed in cold PBS, ground and lysed by electric tissue grinder in RIPA buffer (150 mmol/L sodium chloride, 0.5% Triton-X100, 0.5% sodium deoxycholate, 5 mmol/L EDTA, 0.1% SDS, 50 mmol/L Tris-HCl, pH 7.5) with protease inhibitors cocktail (Roche, USA). Approximately 30 μg total protein from each sample was loaded. Samples were separated on a 10% SDS poly-acrylamide gel and transferred to PVDF membranes (Roche, USA) with electrophoresis blotting transfer apparatus. The membranes were blocked with 5% dehydrated milk for 1.5 h and then incubated with primary antibodies overnight. The next day, the membranes were incubated with horseradish peroxidase-conjugated secondary antibody. The specific signals were detected by ECL plus western blotting detection reagents and X-ray film system.

### Flow cytometry analysis

For immunostaining, cells were fixed by 3.7% formaldehyde for 30 min and permealized by 0.2% Triton X-100 for 5 min. 0.5% donkey serum was used to block the non-specific signals at room temperature for 30 min. Then the samples were incubated sequentially with primary antibody and FITC/APC-Cy7-conjugated secondary antibody (1:400, Life technologies, USA) at room temperature for 1–2 h. BD FACS Verse (BD, USA) equipped with 488 and 647 lasers was used to detect the fluorescence signals for samples. Single cell suspensions went through 40 μm strainer before test. FSC-A and SSC-A parameters were used to exclude the debris and FSC-H, FSC-W, SSC-W parameters were used to exclude the clusters in the cell suspension. IgG control sample was used to set the bottom-line of the positive signals.

### SOX9^+^ BC transplantation clinical trial

A prospective, single-center, non-randomized clinical study was conducted to evaluate the feasibility, safety and efficacy of SOX9^+^ BC transplantation in patients with bronchiectasis. The trial was approved by Southwest Hospital Ethics Committee (Chongqing, China, 2016-Research-#19,ClinicalTrials.gov: NCT02722642), conducted in compliance with Good Clinical Practice (GCP) standard and the most recent version of the Declaration of Helsinki. Two patients who had signed informed consent form were admitted to hospital twice for SOX9^+^ BC isolation and transplantation, respectively. Diagnosis was established based on ATS/ERS guidelines. Patient medical history, vital signs, routine laboratory tests (regular blood counts, biochemical measurements, coagulation test, liver/renal function tests, myocardiozymogram measurements), electrocardiogram, arterial blood gas, pulmonary function tests and HRCT scan were conducted based on standardized clinical SOP of hospital 1 day before and different times after SOX9^+^ BC transplantation. More detailed information for the clinical trial was described in Supplementary Materials.

### Statistics

Block randomization was used to randomize samples/mice into groups of similar sample size. No samples, animals or patients were excluded from all analysis. Statistical analysis was performed by Student’s *t*-tests (two-tail comparisons) or Wilcox test and significant difference was defined as *P* < 0.05. Values in text were presented as means with S.E.M. Microsoft Excel 2011 (Microsoft, USA) or R programming was used for data management, statistical analysis and graph generation. Statistical power analysis was used to ensure adequate sample size for detecting significant difference between samples. The variance is similar between groups that are being statistically compared. All experiments (except the clinical trial) were replicated for at least three times with consistent results in the laboratory. All experimental including the clinical trial outcomes were assessed by at least one blinded participating investigator.

## Electronic supplementary material

Below is the link to the electronic supplementary material.

Supplementary material 1 (PDF 2959 kb)

Supplementary material 2 (PDF 140 kb)
