# Supplementary material for: Regeneration of functional alveoli by adult human SOX9+ airway basal cell transplantation
Source: Protein Cell. 2018 Jan 17;9(3):267–82. doi: 10.1007/s13238-018-0506-y (PMC5829276; doi:10.1007/s13238-018-0506-y)
Supplement: Supplementary file 1 — Supplementary material 1 (PDF 2959 kb) [file 13238_2018_506_MOESM1_ESM.pdf]

Supplementary Figures and Tables

Regeneration of functional alveoli by adult human SOX9^{+} airway basal cell transplantation

Qiwang Ma[1]†, Yu Ma[1,5]†, Xiaotian Dai[2]†, Tao Ren[3]†, Yingjie Fu[4] , Wenbin Liu[1], Yufei Han[1], Yinchuan Wu[1], Yu Cheng[4], Ting Zhang[5], Wei Zuo[1,5,6]*

1. Shanghai Pulmonary Hospital, School of Medicine, Tongji University, Shanghai 200433, China

2. Southwest Hospital, Third Military Medical University of PLA, Chongqing 400038, China

3. Shanghai Jiao Tong University Affiliated Sixth People's Hospital, Shanghai, China #

4. The Institute for Biomedical Engineering and Nano Science, School of Medicine, Tongji University, Shanghai 200029, China

5. Kiangnan Stem Cell Institute, Zhejiang, 311300, China

6. Guangzhou Institute of Respiratory Disease, The First Affiliated Hospital of Guangzhou Medical University, Guangzhou, 510120, China

*Correspondence to: zuow@tongji.edu.cn (Wei Zuo)

† These authors contribute equally to this work.

Supplementary Figure 1

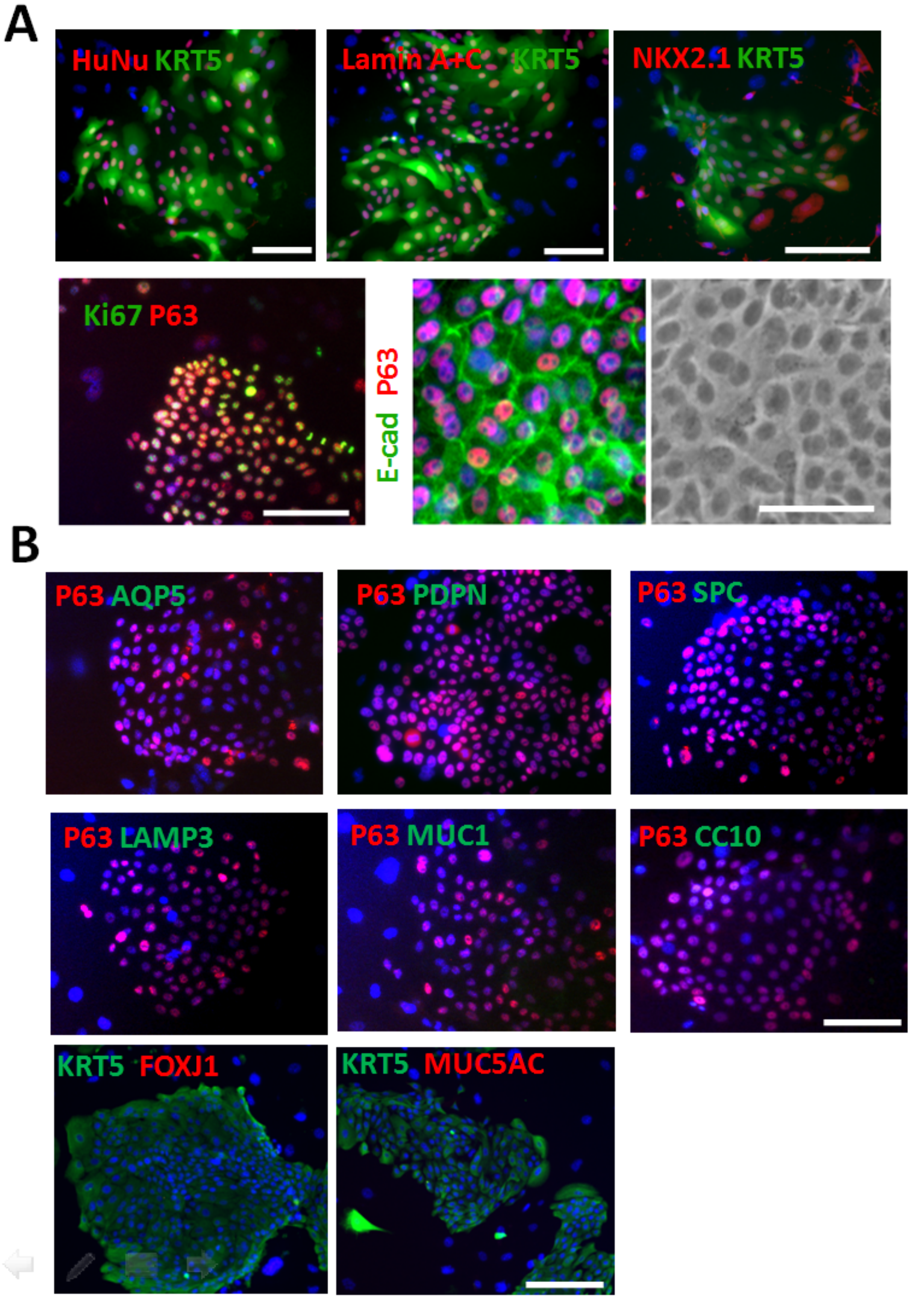

Supplementary Figure 1. Characterization of clonogenic BCs. A, Anti-HuNu, anti-human Lamin A+C, anti-NKX2.1, anti-KI67 and anti-E-Cadherin immunostaining of $KRT5^{+}/P63^{+}$ BC clones. **B**, Immunostaining of BC clones with indicated antibodies and nuclei counterstain. Anti-P63 or anti-KRT5 immunostaining was used to indicate the identity of BC clones. Scale bars, 100 μm.

Supplementary Figure 2.

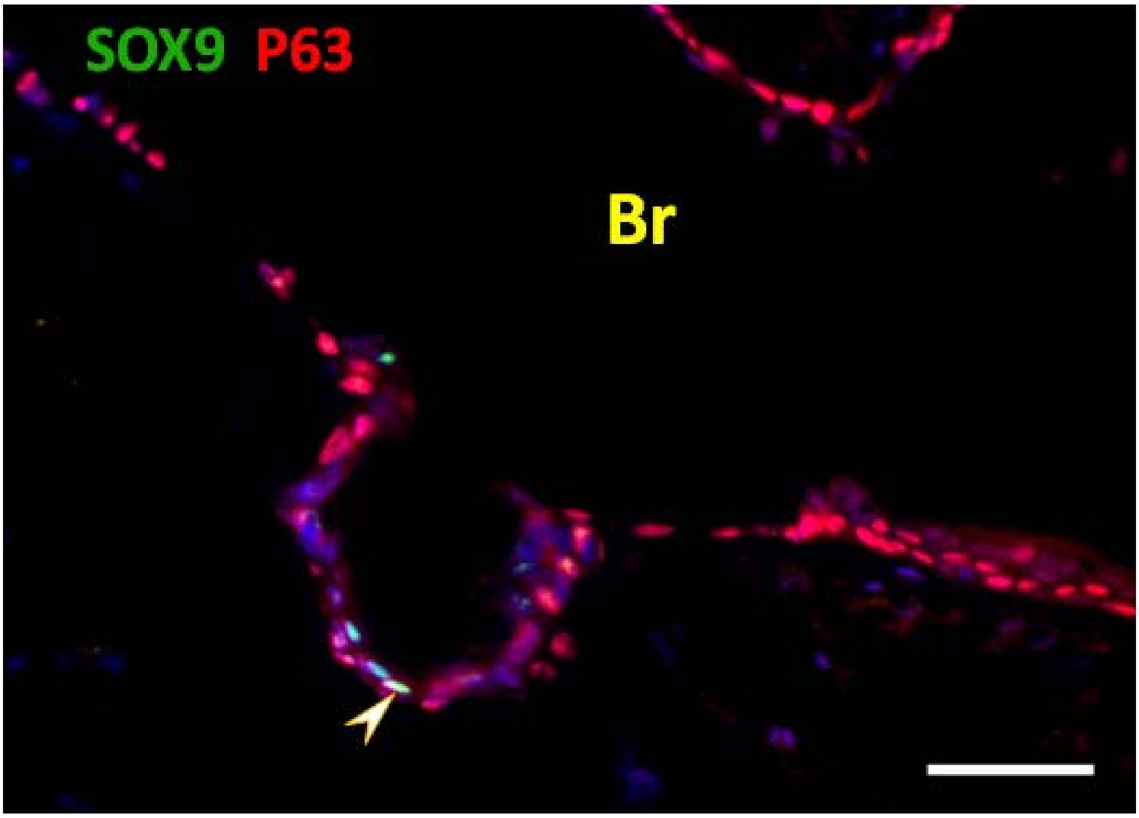

Supplementary Figure 2. **SOX9+ BCs in human lung .** $SOX9^+$ BCs in crypt of 2nd order human airway by anti-SOX9 and anti-P63 immunostaining. Scale bar, 100 μm.

Supplementary Figure 3.

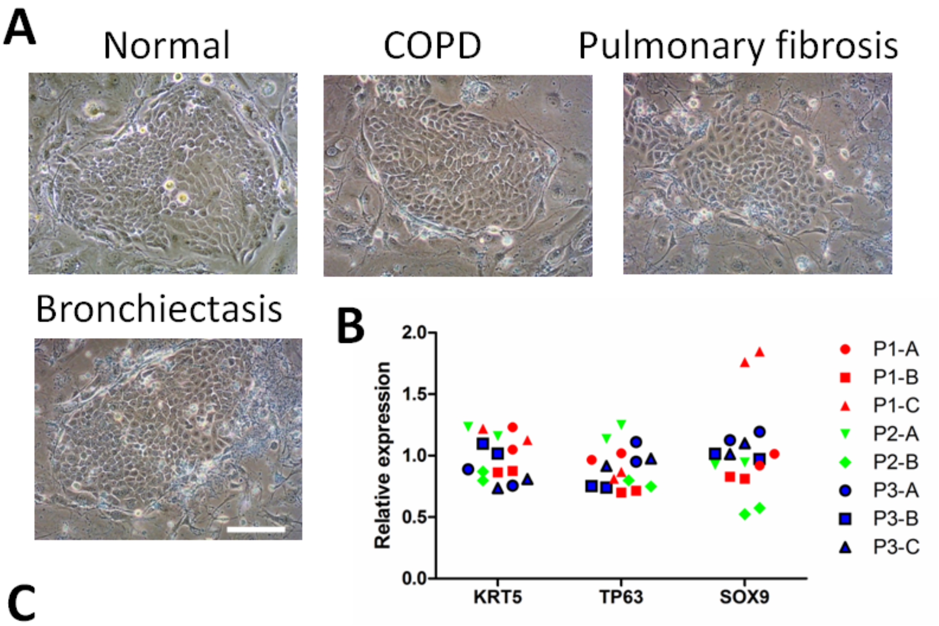

C

Disease Categories	Clone name	Passage 1 (number of clones)	Passage2 (number of clones)
1 (normal)	1-A	152	10,496
	1-B	110	5,796
	1-C	308	22,008
	1-D	118	4,488
	1-E	74	5,416
2 (pulmonary fibrosis)	2-A	188	7,152
	2-B	162	9,784
	2-C	46	3,560
3 (bronchiectasis)	3-A	80	3,668
	3-B	104	5,760
4 (COPD)	4-A	26	89
	4-B	8	200

Supplementary Figure 3. Clonal analysis of SOX9^{+} BCs. A.SOX9^{+} BC clones isolated from persons of different lung disease background. **B**. Diagram showing the relative gene expression of distinct single cell-derived clones. P1, P2 and P3 indicated three individual persons. **C.** Table showing the growth rate of different single cell-derived clones from 4 individual persons**.**

Supplementary Figure 4.

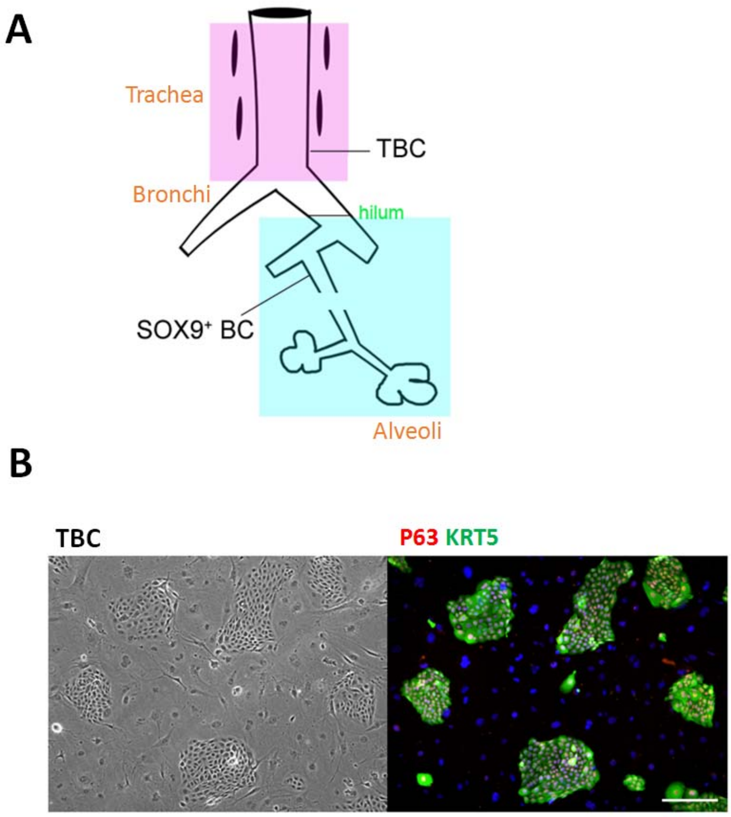

Supplementary Figure 4. Characterization of clonogenic basal cells. A. Diagram showing distinct anatomic locations of isolated tracheal basal cell (TBC) and $SOX9^+$ BC by bronchoscopic brushing. **B**.TBC colonies grown on feeder cells; right, anti-KRT5 and anti-P63 immunostaining of TBC colonies with nuclei counterstain. Scale bar, 100 μm.

Supplementary Figure 5.

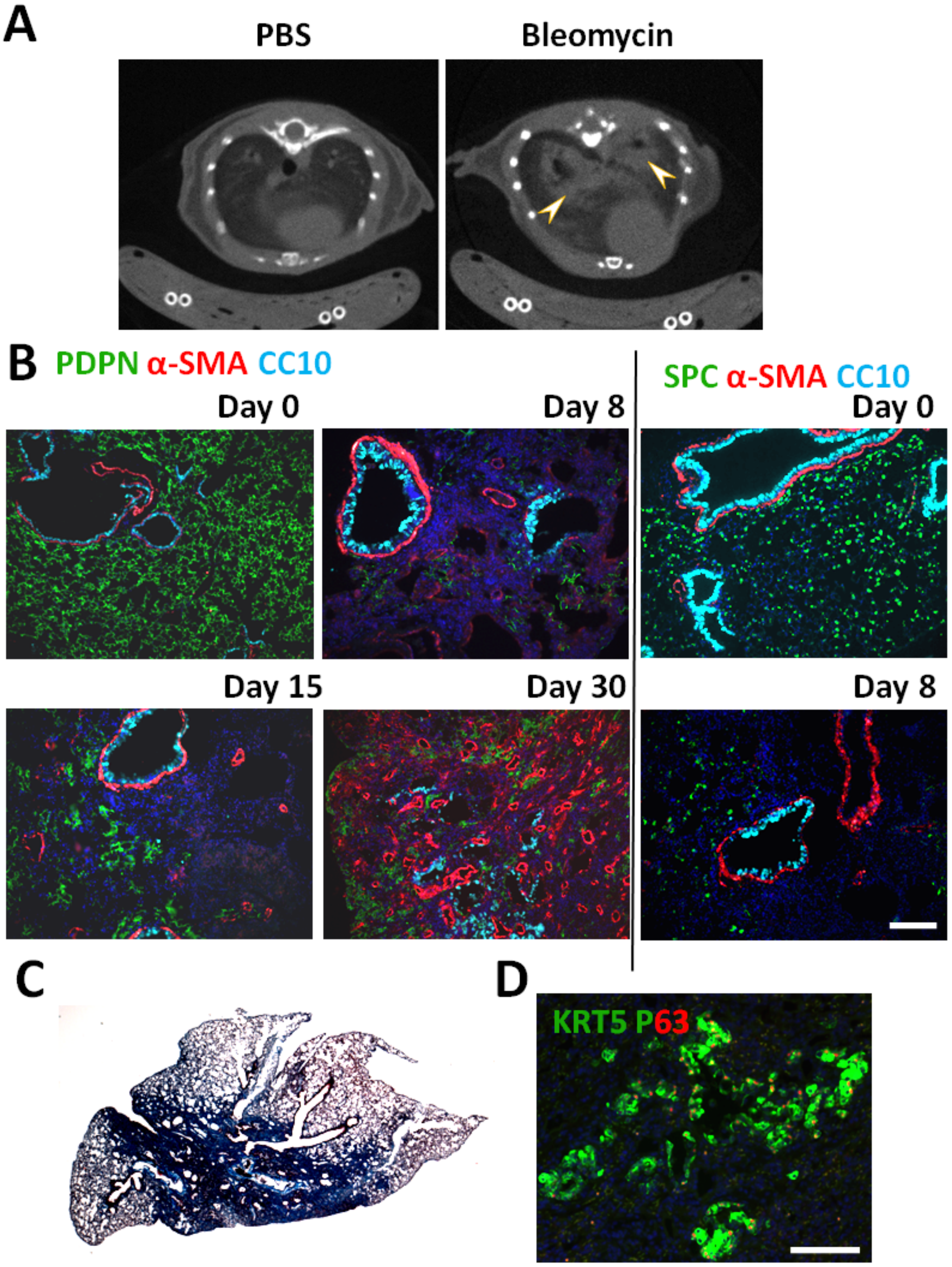

Supplementary Figure 5. Chronic lung injury model for transplantation. A, MicroCT imaging of normal (left) and bleomycin injured (right) mouse lung. Arrowheads indicate lung tissue damage. **B**, Immunostaining of mouse lung after indicated days of bleomycin administration showing loss of lung epithelium marker (PDPN and SPC) and gain of fibrosis marker (a-SMA). **C**, MT (masson trichrome) staining images of mouse lung injured by bleomycin showing large-scale lung fibrosis (30 days post administration). **D**, Anti-Krt5 and anti-p63 immunostaining of mouse lung injured by bleomycin (30 days post administration). Scale bar, 100 μm.

Supplementary Figure 6.

A

Ct value	Mouse	Human	Chimera
Human specific GAPDH	35.2 ± 3.1	14.8 ± 0.2	24.4 ± 0.1

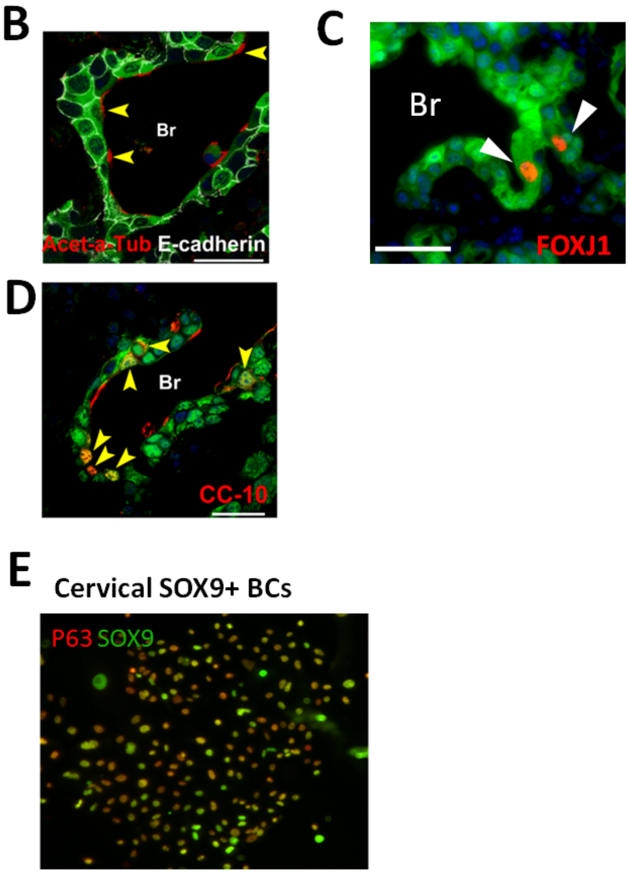

Supplementary Figure 6. Transplantation of human SOX9^{+} BCs into mice generates chimeric lung. A. Normalized Ct values of qPCR reaction with indicated human-specific GAPDH primer pairs. In the sample of mouse lung, both high Ct and abnormal melt curve was observed for GAPDH. n=3, biological replicates. **B-D.** Immunofluorescent images showing regenerated ciliated cells (Acetylated-Tubulin+, FOXJ1+) and Club cells (CC10+) by SOX9+ BC transplantation. Scale bar: 10 μm. **E**.Clonogenic BCs isolated from human cervix epithelium obtained by biopsy, characterized by immunostaining and labeled by GFP.

Supplementary Figure 7.

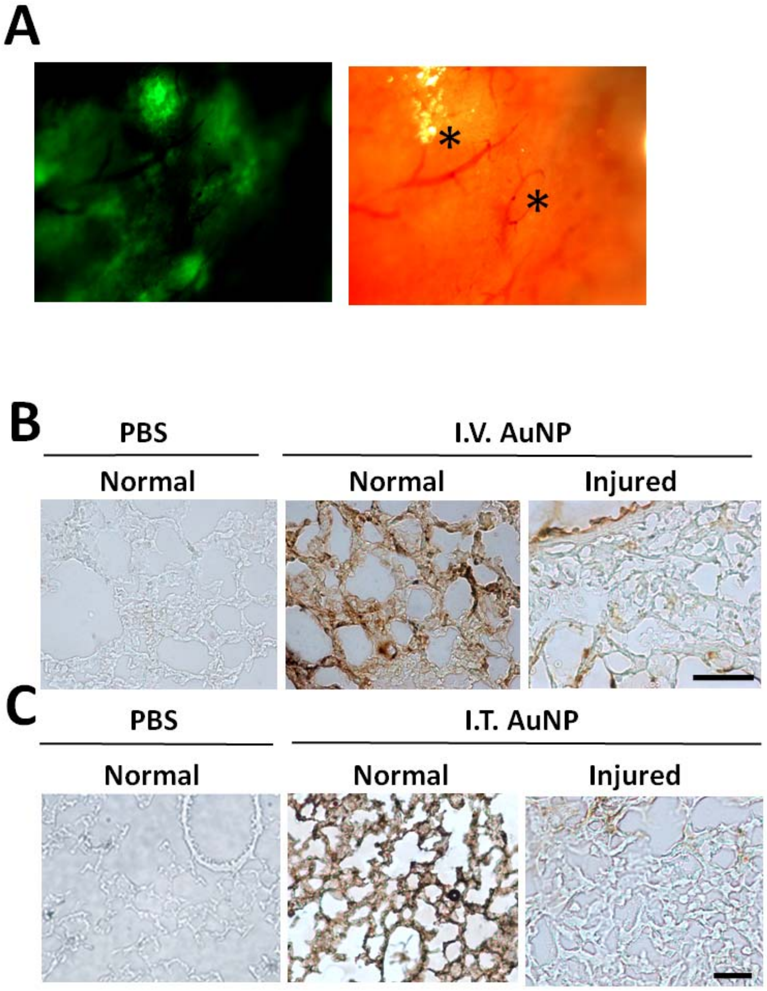

Supplementary Figure 7. Functional epithelium-vasculature interaction in regenerated lung. A, Photo-cleared mouse lung after GFP^{+}SOX9^{+} BCs transplantation was subjected to direct fluorescence imaging (left) and blood vessel visualization (right, asterisks) in the same region under stereomicroscope. **B**, Bright-field imaging of intravenously (I.V.) delivered AuNP (brown) of normal or injured region of mouse lung. Rare signal detected in injured region indicated deficiency of blood-gas exchange function. **C**, Bright-field imaging of intratracheally (I.T.) delivered AuNP (brown) of normal or injured region of mouse lung. Rare signal detected in injured region indicated deficiency of inhale function. Scale bar, 100 mm.

Supplementary Figure 8.

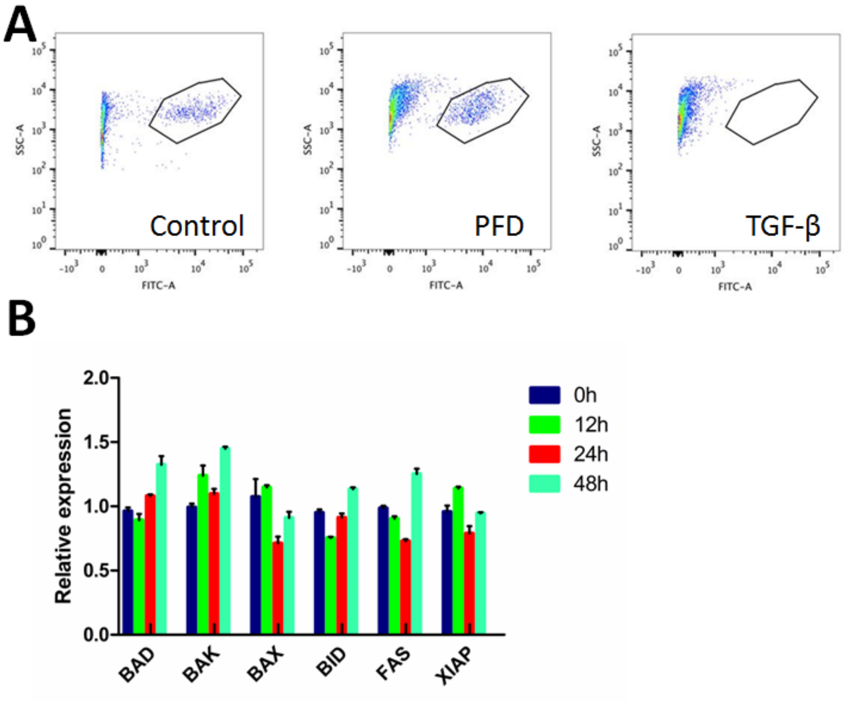

Supplementary Figure 8. TGF-β modulates SOX9⁺ BC proliferation. A, Representative flow cytometry analysis of GFP+ cells in SOX9⁺ BC transplanted mouse lung with Pirfenidone or TGF-β treatment, respectively. **B,** qPCR showing apoptosis-related gene expression level of SOX9+ BCs with 10 ng/ml TGF-β treatment for indicated hours. n=3, biological replicates. Error bars, S.E.M. n.s, not significance.

Supplementary Table 1. qPCR primer information.

Primer	Primer sequence (5' – 3')
AQP5F	ACCCTTCCTCAAGAGCTGAAG
AQP5R	GGTAGCCCCCACTCTAAACAC
HOPXF	CAGGGACGAGAAGATAGTGAC
HOPXR	CTGAAAGTATGCCGAGGTT
PDPNF	GTCCACGCGCAAGAACAAAG
PDPNR	GGTCACTGTTGACAAACCATCT
SFTPBF	CTGTCTCAGCTCAACCACAGTC
SFTPBR	GTAAAGCAGTGGCTGGTTTTTC
LAMP3F	GCCTTTGATTTTGAAGATGACC
LAMP3R	GGGCAACAATTAGATTCTCTGG
SCGB1A1F	TCATGGACACACCCTCCAGTTATGAG
SCGB1A1R	TGAGCTTAATGATGCTTTCTCTGGGC
SCGB3A2F	GACAACATTCTTCCCTTTAT
SCGB3A2R	CTCCAGCAGTTTCTTCACA
MUC1F	CCACCCATTTCACCACCAC
MUC1R	AACCCGTAACAACTGTAAGCAC
P15 F	GACACTCACCATGAAGCGAAACA
P15 R	TCGTAGCCACCAGGTCCAGTC
P21 F	CGATGGAACTTCGACTTTGTCA
P21 R	GCACAAGGGTACAAGACAGTG
CCNA1 F	ACATGGATGAACTAGAGCAGGG
CCNA1 R	GAGTGTGCCGGTGTCTACTT
CCNE2 F	TCAAGACGAAGTAGCCGTTTAC
CCNE2 R	TGACATCCTGGGTAGTTTTCCTC
CDK4 F	ATGGCTACCTCTCGATATGAGC
CDK4 R	CATTGGGGACTCTCACACTCT
PCNA F	ACACTAAGGGCCGAAGATAACG
PCNA R	ACAGCATCTCCAATATGGCTGA
ACTBF	TTTGAATGATGAGCCTTCGTGCCC
ACTBR	GGTCTCAAGTCAGTGTACAGGTAAGC
GAPDHF	AGTATGACAACAGCCTCAAGAT
GAPDHR	GTCCTTCCACGATACCAAA
ACTN1 F	AACATCGAAGAGGACTTCCG
ACTN1 R	CAAGCGTTCACCTGAGATGAC

#

Supplementary Table 2. Information of SOX9+ BC donors

#

ID	Age	Sex	Ethnic	Type of disease	Degree of respiratory disorders
1	65	male	Chinese	Normal	NA
2	57	male	Chinese	Normal	NA
3	24	male	Spanish	Normal	NA
4	43	female	Chinese	Normal	NA
5	30	female	Chinese	Normal	NA
6	43	female	Chinese	Bronchiectasis	moderate
7	57	male	Chinese	Bronchiectasis	extremely severe
8	57	male	Chinese	Chronic Obstructive Pulmonary Disease	moderate
9	60	male	Chinese	Chronic Obstructive Pulmonary Disease	moderate
10	72	male	Chinese	Chronic Obstructive Pulmonary Disease	severe
11	65	male	Chinese	Interstitial Lung Disease	severe
12	36	male	Chinese	Interstitial Lung Disease	moderate
13	53	male	Chinese	Interstitial Lung Disease	mild
14	45	male	Chinese	Interstitial Lung Disease	moderate
15	42	female	Chinese	Interstitial Lung Disease	extremely severe

#
