# Supplementary material for: Regeneration of functional alveoli by adult human SOX9+ airway basal cell transplantation
Source: Protein Cell. 2018 Jan 17;9(3):267–82. doi: 10.1007/s13238-018-0506-y (PMC5829276; doi:10.1007/s13238-018-0506-y)
Supplement: Supplementary file 2 — Supplementary material 2 (PDF 140 kb) [file 13238_2018_506_MOESM2_ESM.pdf]

# Clinical Trial Information

## Regeneration of functional alveoli by adult human $SOX9^+$ airway basal cell transplantation

Qiwang Ma[1]†, Yu Ma[1,5]†, Xiaotian Dai[2]†, Tao Ren[3]†, Yingjie Fu[4] , Wenbin Liu[1], Yufei Han[1], Yinchuan Wu[1], Yu Cheng[4], Ting Zhang[5], Wei Zuo[1,5,6]*

1. Shanghai Pulmonary Hospital, School of Medicine, Tongji University, Shanghai 200433, China
2. Southwest Hospital, Third Military Medical University of PLA, Chongqing 400038, China
3. Shanghai Jiao Tong University Affiliated Sixth People's Hospital, Shanghai, China
4. The Institute for Biomedical Engineering and Nano Science, School of Medicine, Tongji University, Shanghai 200029, China
5. Kiangnan Stem Cell Institute, Zhejiang, 311300, China
6. Guangzhou Institute of Respiratory Disease, The First Affiliated Hospital of Guangzhou Medical University, Guangzhou, 510120, China

*Correspondence to: zuow@tongji.edu.cn (Wei Zuo)

† These authors contribute equally to this work.

**General trial design:**

This trial aims to treat bronchiectasis by autologous basal cell transplantation. In this single-center, non-randomized, self controlled, phase I/II clinical trial, patients' own basal cells from bronchioles will be grown in GMP facility before injected to the damaged part of lung tissue. In total 20 participants are estimated to be enrolled in the trial since March 2016. The primary endpoints of this trial include: 1) safety of the treatment; 2) Improvement of HRCT imaging after treatment. The secondary endpoints include: 1) Elevation rate of DLCO/VA after treatment; (2) Elevation rate of FEV1 after treatment; (3) Elevation rate of FVC after treatment.

**Inclusion criteria of this clinical trial is:**

- Age 18~75 years;
- Diagnosed as bronchiectasis by HRCT scan;
- At least 6 lung segments are affected;
- Clinically stable for at least 2 weeks prior to study entry;
- Be able to tolerate bronchoscopy.

**Exclusion criteria of this clinical trial is:**

- Women of child bearing age at the stage of pregnancy or lactation, or those without taking effective contraceptive measures.
- Subjects with syphilis or HIV positive antibody.
- Subjects with any malignancy.
- Subjects suffering from any of the following pulmonary diseases: active tuberculosis, pulmonary embolism, pneumothorax, multiple huge bullae, uncontrolled asthma, acute exacerbation of chronic bronchitis or extremely severe COPD.
- Subjects suffering from other serious diseases, such as diabetes, myocardial infarction, unstable angina, cirrhosis, and acute glomerulonephritis.
- Subjects with leukopenia (WBC less than 4x10^9 / L) or agranulocytosis (WBC less than 1.5x10^9 / L or neutrophils less than 0.5x10^9 / L) caused by any reason.
- Subjects with severe renal impairment, serum creatinine> 1.5 times the upper limit of normal.
- Subjects with liver disease or liver damage: ALT, AST, total bilirubin> 2 times the upper limit of normal.
- Subjects with a history of mental illness or suicide risk, with a history of epilepsy or other central nervous system disorders.

- Subjects with severe arrhythmias (such as ventricular tachycardia, frequent superventricular tachycardia, atrial fibrillation, and atrial flutter, etc.) or cardiac degree II or above conduction abnormalities displayed via 12-lead ECG.
- Subjects with a history of alcohol or illicit drug abuse.
- Subjects accepted by any other clinical trials within 3 months before the enrollment.
- Subjects with poor compliance, difficult to complete the study.
- Any other conditions that might increase the risk of subjects or interfere with the clinical trial.

**Time table of SOX9+ BC transplantation treatment and observation**

| Operations / Time | -5~-3 weeks | -1~0 day | 3 days | 30 days | 90 days | 1 year |
|---|---|---|---|---|---|---|
| Sign of informed consent | X | | | | | |
| Inclusion/exclusion criteria screen | X | X | | | | |
| Medical history inquiry | X | X | X | X | X | X |
| Symptom inquiry | X | X | X | X | X | X |
| Body examination | X | X | X | X | X | X |
| Vital signs examination | X | X | X | X | X | X |
| Body weight examination | X | X | | X | X | X |
| Regular blood test | X | X | X | X | X | X |
| Pregnancy test* | | X1 | | | | |
| Blood biochemical test | | X | X | X | X | X |
| Blood coagulation test | X | X | | | | |
| CRP | | X | X | X | X | X |
| Myocardiozymogram | | X | X | X | X | X |
| VDRL，HIV test | X | | | | | |
| electrocardiogram | X | X | X | X | X | X |
| Chest HRCT | | X | X | X | X | X |
| Bronchoscopic brushing | X | | | | | |
| $SOX9^{+}$ BC transplantation | | X | | | | |
| Blood-gas analysis | | X | X | X | X | X |
| Spriometry | | X2 | | X | X | X |
| 6MWT | | X | | X | X | X |
| SGRQ score | | X | X | X | X | X |
| Combined medicine | X | X | X | X | X | X |
| Adverse events evaluation | | | X | X | X | X |
| Compliance of patients evaluation | | | X | X | X | X |

*18~45 female only

**Procedure of SOX9$^+$ BC transplantation:**

1) The clinical trial was approved by Southwest Hospital Ethics Committee (Chongqing, China) and registered in ClinicalTrial.gov database (NCT02722642). Patients diagnosed as bronchiectasis by ATS/ERS criteria were enrolled into the clinical trial group according to the inclusion/exclusion criteria. All enrolled patients signed an informed consent form. In the consent form, patients were informed of the research background, biological properties of SOX9$^+$ basal cells, procedure of treatment, unsuitable participants, possible benefits and potential risks. The patients were free to quit the clinical trial anytime and all cost related to the trial was covered by the researcher. All medical records of patients were kept confidential unless required to uncover.
2) To obtain SOX9$^+$ BCs, the bronchoscopic procedure for sampling was performed by board-certified respiratory physicians using a flexible fiber-optic bronchoscope (Olympus, Japan). Before the bronchoscopy, oropharyngeal and laryngeal anesthesia was obtained by administration of 2 mL of nebulized 4% lidocaine, followed by 1 mL of 2% topical lidocaine sprayed into the patient's oral and nasal cavities. After the bronchoscope was advanced through the vocal cords, 2 mL of 2% lidocaine solution was instilled into the trachea and both main bronchi through the working channel of the bronchoscope. Then a disposable 2-mm brush was advanced through the working channel of the fiberoptic bronchoscope and used to collect airway epithelial cells by gently gliding the brush back and forth 1 or 2 time in random regions of trachea or 3~4 order bronchi in the right or left lobe. Brushed tissues were briefly digested by 0.05% trypsin and then used for SOX9$^+$ BC culture.
3) Isolated SOX9$^+$ BCs were cultured on feeder cells and then shifted to feeder-free culture condition as described in the method part of manuscript according to Good Manufacture Practice (GMP) by qualified operators with GCP (Good Clinical Practice) certificate. Quality of clinically used cells (bacteria, endotoxin and pyrogen-free) was confirmed by International Laboratory Accreditation Cooperation- Mutual Recognition Arrangement (ILAC-MRA) recognized third-party organization. Expression of SOX9$^+$/P63$^+$/KRT5$^+$ markers, normal cell karyotype, cell in vitro differentiation capability, free of antibiotics remaining and free of animal-derived virus were also examined. Cell viability (>95%) was examined one hour before transplantation. SOX9+ BCs passing the quality control were harvested, washed by saline solution and counted by automatic cell counter. $10^6$/kg body weight cells were suspended in 10.5 mL sterile 0.9% NaCl and shipped on ice from GMP facility to hospital by airplane.
4) Cell suspension was pre-warmed to 37 degree 15 minutes before use. After local anesthesia of patients, bronchoalveolar lavage was performed with 50mL saline. After lavage and when the oxygen saturation of patient reached >92%, fibrotic bronchoscopy was guided into the lobular or segmental airways and cells were instilled into lobes. The amount of cell suspensions instilled into each lobe was adjusted based on their damaged level based on CT result. After transplantation,

the patient was advised to fast and avoid coughing for two hours. Patient was discharged from hospital 3 days after transplantation.
